# Single-port vs multi-port laparoscopic appendectomy in acute appendicitis: a systematic review

**DOI:** 10.1007/s00423-025-03923-1

**Published:** 2025-12-18

**Authors:** En Qing Lim, Aaron Jun Ket Lim, Adil Lakha, Zeeshan Razzaq

**Affiliations:** 1 School of Medicine, University of Dundee, Dundee, Scotland, UK; 2 Department of Surgery, Cork University Hospital, Cork, Republic of Ireland; 3 Universiti Teknologi Malaysia, Johor, Malaysia; 4 Nuffield Department of Surgical Sciences, University of Oxford, Oxford, UK; 5 Oxford University Hospitals NHS Foundation Trust, Oxford, UK; 6 Consultant General Surgeon, St. Luke’s General Hospital, Kilkenny, Republic of Ireland

**Keywords:** Single-port laparoscopic surgery (SPLS), Multi-port laparoscopic surgery (MPLS), Laparoscopic appendectomy, Perforated appendicitis, Emergency surgery, Appendicitis, Appendectomy

## Abstract

**Background/aims:**

Single-port laparoscopic surgery (SPLS) is a promising alternative to multi-port laparoscopic surgery (MPLS) for emergency appendectomy, with potential advantages in cosmesis and recovery. However, its role remains uncertain, particularly in complex cases. This systematic review and meta-analysis evaluates SPLS's safety, effectiveness, and recent advancements to inform clinical decisions and practice.

**Methods:**

A systematic search of PubMed, Embase, and Cochrane Library identified studies comparing SPLS with MPLS in emergency appendectomy. Outcomes included conversion to open surgery, operative time, postoperative complications, length of hospital stay, pain outcomes, and cosmetic satisfaction. Data synthesis followed PRISMA guidelines using a random-effects model.

**Results:**

Eleven studies, including randomized controlled trials and cohort studies, were included. No significant differences were observed in conversion rates (OR 1.78, 95% CI: 0.63–5.03), complication rates (OR 0.96, 95% CI: 0.63–1.46), operative time (mean difference 2.93 min, 95% CI: − 3.17 to 9.02), length of stay (mean difference − 0.23 days, 95% CI: − 0.62 to 0.15), or postoperative pain (mean difference − 0.24, 95% CI: − 0.96 to 0.49). Substantial heterogeneity was present for operative time (I^2^ = 91%), length of stay (I^2^ = 92%), and pain (I^2^ = 80%). Cosmetic satisfaction generally favoured SPLS, although assessment methods varied considerably.

**Conclusion:**

While SPLS appears to be a safe and feasible alternative to MPLS for emergency appendectomy, current evidence does not support definitive equivalence across all outcomes due to significant heterogeneity, small study sizes, and inconsistent measurement tools. Future large-scale randomized trials are necessary to clarify SPLS’s role, especially in complex cases and high-risk populations.

## Introduction

The development of minimally invasive surgical techniques revolutionized the management of acute appendicitis. Laparoscopic appendectomy (LA) has now been widely accepted as the standard of care due to improved outcomes when compared with open surgery [1]. Conventional multi-port laparoscopic surgery (MPLS) involves three small incisions, typically at the umbilicus, suprapubic area, and lower abdomen, to accommodate surgical instruments. MPLS has consistently demonstrated safety, efficacy, and faster recovery times compared to open appendectomy [2]. The introduction of single-port laparoscopic surgery (SPLS) is an innovative approach to improve patient outcomes through further reduction in the number of port incision sites into a single-entry port. Potential benefits include reduced surgical trauma and improved cosmesis. In contrast, poor ergonomics, technical challenges in understanding the procedure, and perceived lack of versatility to deal with intra-operative complications represent possible drawbacks of SPLS.

Previous literature has demonstrated that SPLS is a feasible and safe alternative to MPLS for the management of acute appendicitis, with favourable outcomes in terms of reduced postoperative pain and cosmetic results [3–5]. SPLS has shown especially good efficacy in some complex clinical situations, such as interval appendectomy for perforated appendicitis and appendicitis during pregnancy [3, 6].

However, there is limited evidence directly comparing SPLS with MPLS. Although SPLS has been shown to have some advantages in aesthetic satisfaction and reduction of postoperative pain [7, 8], continuing controversy surrounds its surgical time, complication rates and economic feasibility [5, 9]. These discrepancies further support the need to better understand the relative benefit of SPLS and specifically, its suitability in urgent and complex surgical settings.

Previous meta-analyses, including a 2024 Cochrane review by Irfan et al. [10], have investigated the efficacy of single-incision laparoscopic appendectomy. While their findings also support SPLS as a viable alternative to MPLS, they excluded more recent RCTs and did not focus on emergency or complex cases, such as perforated appendicitis. Other reviews, such as those by Aly et al. [9] and Zaman et al. [8], were limited in scope by excluding high-risk populations or lacked quantitative synthesis. This study aims to address these gaps by incorporating updated literature, including 2023–2024 trials, and evaluating a broader range of clinical scenarios.

## Methods

**Fig. 1:**
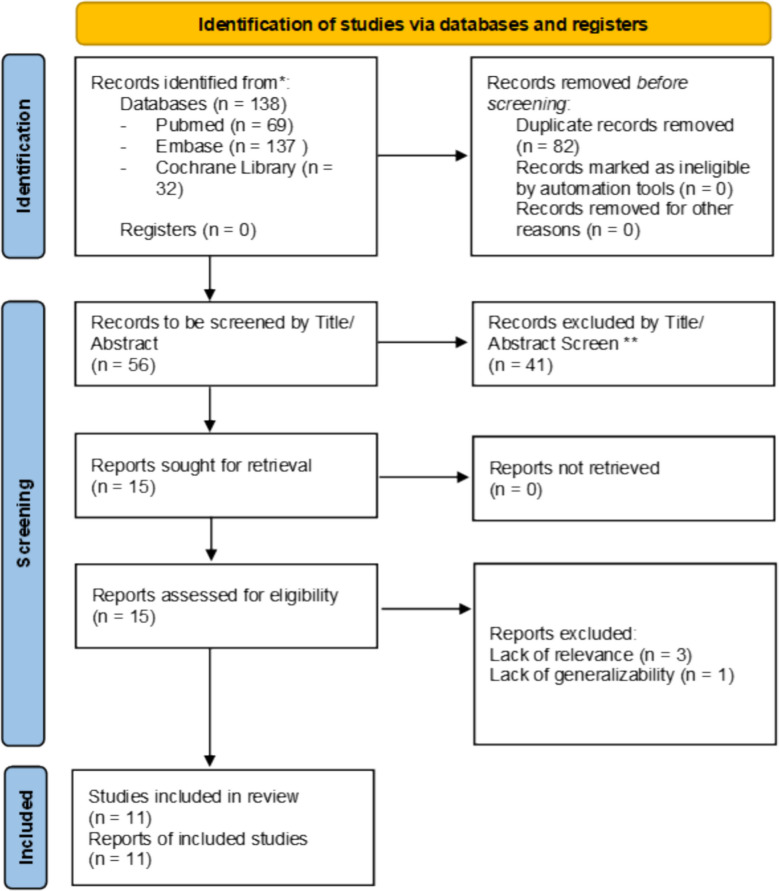
PRISMA flowchart

This systematic review was performed in accordance with the Preferred Reporting Items for Systematic Reviews and Meta- Analysis (PRISMA) statement [11] (Fig. 1). The review was prospectively registered on PROSPERO, CRD42025642574.

We conducted a systematic search of three databases, including PubMed, Embase, and Cochrane Library, using the following search terms as Medical Subject Headings (MeSH) terms or in all fields:

- “Single-port laparoscopic surgery” OR “SPLS”
- AND “Multi-port laparoscopic surgery” OR “MPLS”
- AND “Appendectomy” OR “Emergency surgery”
- AND “Postoperative outcomes” OR “Pain” OR “Cosmetic satisfaction.”

Studies from inception of each database up until July 31, 2024 of which only those available in English and full text were considered for inclusion.

## Inclusion criteria

Studies involving adults (≥ 18 years) undergoing SPLS or MPLS for emergency appendectomies, with no limitations on comorbidities or complexity of appendicitis (e.g., perforated appendicitis), or study location.

Given the heterogeneity of surgical practice globally, we opted for inclusive criteria to reflect real-world application of SPLS across varied clinical settings. However, we acknowledge this may introduce clinical heterogeneity, which is addressed in sensitivity and subgroup analyses.

## Exclusion criteria

We excluded studies that involved non-human subjects and all paediatric populations.. Case reports, editorials, commentaries, letters to the editor and replies were all excluded.

## Selection of studies

Two review authors (EQL and AJKL) independently screened titles and abstracts of the articles retrieved from the three databases. Then, irrelevant studies and duplicates were excluded. Any discrepancies were discussed until consensus reached, and any disagreement was resolved by a third, independent author.

The remaining articles were then reviewed based on the inclusion and exclusion criteria set beforehand, with both review authors (EQL and AJKL) conducting this step independently once again.

A final list of articles was produced, and any disagreements arising were resolved through discussion by two of the authors (EQL and AJKL); any discrepancies were discussed until consensus reached, and any disagreement was resolved by a third, independent author (ZR). Two review authors (EQL and AJKL) independently collected data from eligible studies, and disagreements were resolved with discussion and involvement of the senior author (ZR) if needed.

All data extracted were tabulated and recorded on a Microsoft Excel sheet.

## Quality assessment

Two review authors (EQL and AJKL) worked independently in assessing the quality of studies and results were compared. All data was cross-verified and any differences in scores was resolved via a thorough discussion between the authors (EQL, AJKL, AL, ZR).

The Newcastle–Ottawa Scale (NOS) was used to assess the quality of included studies identified from our search protocol [12]. The NOS assesses studies in three domains, including the selection of groups, comparability between groups, and the outcomes of cohort studies. It utilizes a point-based system and each study is given a point between 0 to 9. Studies with scores of 7 or greater were considered as high quality (Table 2).

**Fig. 2:**
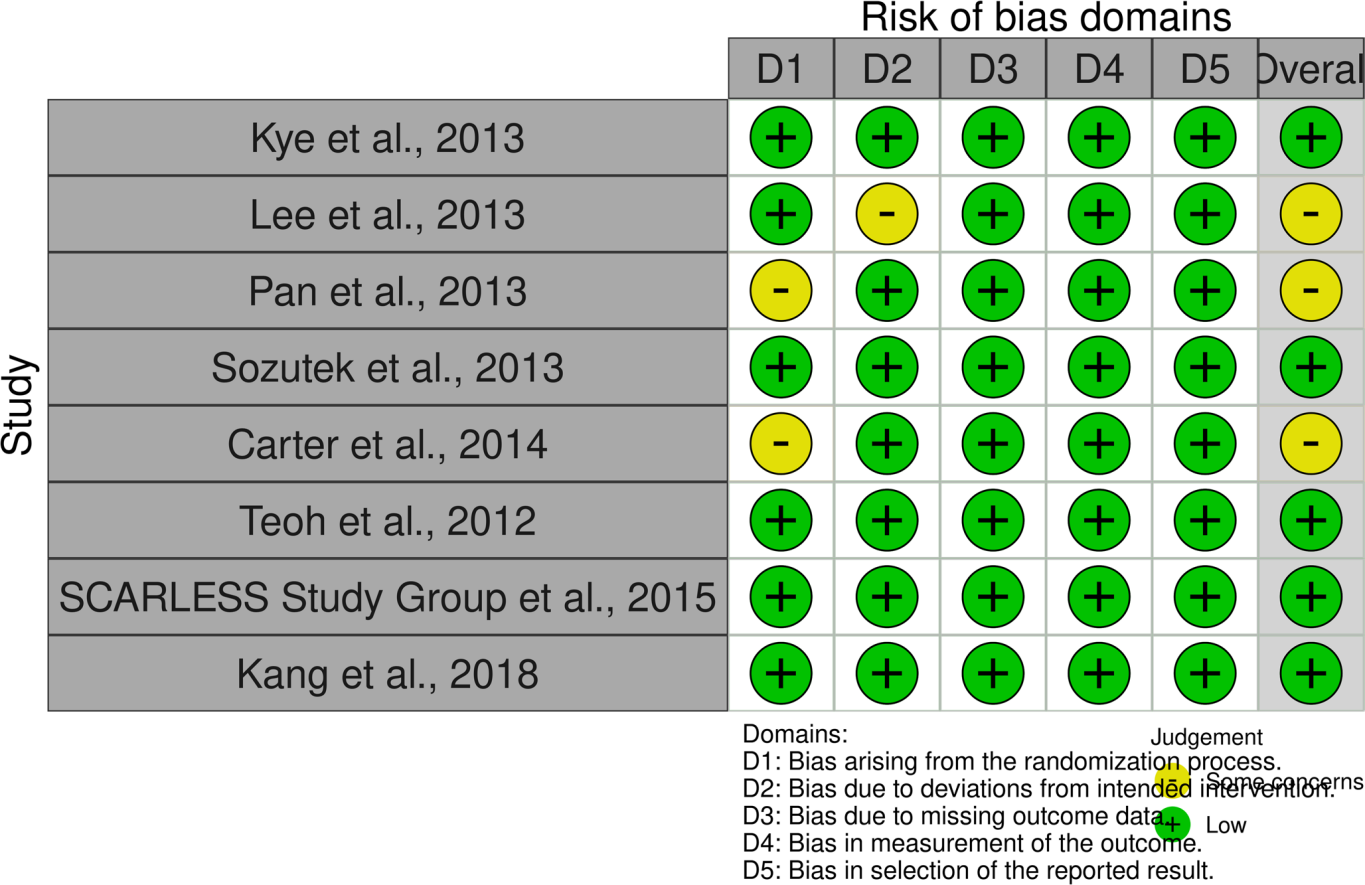
Results of RoB-2 risk-of-bias assessment of included RCTs

The RoB-2 tool was employed to assess the risk of bias in all randomized controlled trials included in this review. It evaluates multiple domains such as study design, execution, and reporting of outcomes to classify the risk of bias as low, moderate, or high (Fig. 2)[13].

Both tools conform to methodological standards outlined by the Agency for Healthcare Research and Quality (AHRQ) for systematic reviews[14].

Review authors (EQL and AJKL) worked independently in assessing the quality of studies and results were compared. Any differences in scores was resolved via a thorough discussion between the authors.

## Data analysis

Continuous variables were presented as the mean and standard deviation (SD), while categorical variables were expressed as percentages. Where standard deviations were not reported, we contacted the corresponding authors to request additional information. In cases where no response was received, as in Lee et al. [15] which reported length of hospital stay as mean and range [15], we applied the method proposed by Walter and Yao [16] to estimate standard deviation from reported ranges or interquartile ranges.

A meta-analysis was conducted to determine the secondary outcomes between SPLS and MPLS. Since substantial heterogeneity among the studies was expected, a random-effects model was applied. The I^2^ statistic was used to determine the magnitude of variability between included studies, with a value of more than 75% denoting considerable heterogeneity. A Z-test was also conducted to estimate the overall effect with the level of statistical significance at *p < *0.05.

All statistical analyses were carried out using Review Manager (RevMan) [Computer program], Version 5.4, The Cochrane Collaboration, 2020.

## Results

The literature search identified 138 articles for review. All articles were accessed enabling assessment throughout the selection process, resulting in 11 studies included (Fig. 1). 8 randomised controlled trials, and 3 retrospective cohort studies were included, with study characteristics and results summarized in Tables 1, 2 and 3, respectively.

**Table 1:** Characteristics of included studies. Continuous variables are presented as mean ± SD or median (range)

Study characteristics						
Author(s), Year of publication	Study design	Age, SPLS; MPLS (years, Mean ± SD or Range)	Sex (Male, n [%])	Country of origin	Sample size, n (SPLS; MPLS)	Type of intervention
Liu et al. [5]	Retrospective Cohort Study	34.3 ± 10.5; 41.6 ± 14.5	16 (29.6%); 130 (53.7%)	China	296 (54; 242)	Single-Port vs Three-Port
Kye et al. [7]	Randomized Controlled Trial	27.55 ± 12.40; 29.20 ± 13.98	26 (51%); 26 (51%)	South Korea (single center)	102 (51; 51)	Single-Incision vs Three port
SCARLESS Study Group et al., 2015 [17]	Randomized Controlled Trial	27 (19–45); 32 (21–38)	20 (51%); 20 (53%)	United Kingdom (single center)	(39, 38)	Single-port vs conventional three port
Carter et al. [18]	Randomized Controlled Trial	34 ± 11; 35 ± 12	19 (51%); 24 (63%)	USA (single center)	(37; 38)	Single-port vs conventional three port
Lee et al. [15]	Randomized Controlled Trial	28.4(range 16–76); 28.5 (range 16–78)	64 (55%); 68 (60%)	Korea (single center)	(116; 113)	Single-port vs conventional three port
Pan et al. [19]	Randomized Controlled Trial	34.1 ± 14.5 (16–72); 34.9 ± 14.9 (16–82)	24 (57%); 20 (48%)	China (single center)	(42; 42)	Single-port vs conventional three port
Sozutek et al. [20]	Randomized Controlled Trial	30.6 ± 12.4; 30.0 ± 11.0	12 (48%); 7 (28%)	Turkey (single center)	(25; 25)	Single-port vs standard three port vs open appendectomy
Teoh et al. [21]	Randomized Controlled Trial	39.2 ± 15.5; 40.7 ± 15.7	58 (59%); 59 (61%)	Hong Kong multicentre	(98; 97)	Single-Site Access (LESS) appendectomy vs Conventional Three-Port Laparoscopic Appendectomy (TPLA)
Kim et al. [22]	Retrospective cohort study with propensity score matching (PSM)	40.2 ± 13.6; 36.0 ± 19.2	18 (51%), 22 (63%)	Korea	(35; 35)	Single-Incision Laparoscopic Appendectomy (SILA) vs Conventional Laparoscopic Appendectomy (CLA)
Kang et al. [23]	Randomized Controlled Trial	32.4 ± 15.4; 29.6 ± 14.7	52 (57.8%); 54 (60.0%)	South Korea (single- center)	(90; 90)	SPLS vs MPLS
Assali et al. [24]	Retrospective Cohort Study	39.2 ± 16.0; 40.5 ± 16.4	62 (48.4%); 458 (48.7%)	United States	1069 (128; 941)	Single-Port Laparoscopic Appendectomy (SPLA) versus Multi-Port Laparoscopic Appendectomy (MPLA)

**Table 2:** Outcomes from assessment of risk bias of cohort studies using the Newcastle–Ottawa Scale

First author, year of publication	Total quality score (Max 9)	Selection (Max 4)	Comparability (Max 2)	Exposure/Outcome (Max 3)
Liu et al. [5]	7	4	1	2
Kim et al. [22]	6	3	1	2
Assali et al. [24]	6	3	1	2

**Table 3:** Results of individual studies. Continuous variables are presented as mean ± SD or median (range)

Results of individual studies						
Author(s), Year of publication	Conversion rates to open surgery, SPLS; MPLS (%)	Operative time, SPLS; MPLS (min, Mean ± SD)	Length of hospital stay, SPLS; MPLS (days, Mean ± SD)	Postoperative pain (VAS score)	Cosmetic satisfaction (%)	Postoperative complications, SPLS; MPLS (n, %)
Liu et al. [5]	N/A	86.8 ± 26.5; 80.0 ± 44.4	1.3 ± 0.7; 3.6 ± 3.0	N/A	N/A	2 (3.7%); 17 (7.0%)
Kye et al. [7]	1 (1.96%); 1 (1.96%)	37.00 ± 15.46; 38.45 ± 15.26	2.78 ± 1.22; 2.83 ± 1.29	3.22 ± 1.22; 3.90 ± 1.46	N/A	2 (3.9%); 2 (3.9%)
SCARLESS Study Group et al., 2015 [17]	1 (3%); 2 (5%)	74 ± 23; 89 ± 37	3.0 ± 2.8; 2.8 ± 4.4	N/A	Higher patient satisfaction in SPILS group (not quantified);	3 (8%); 5 (13%)
Carter et al. [18]	1 (3%); 0 (0%)	54 ± 17; 38 ± 12	1.4 ± 0.8; 1.6 ± 1.8	Pain in first 12 h: 4.4 ± 1.6; 3.5 ± 1.5	Overall Body Satisfaction 3.5 ± 1.0; 3.7 ± 0.6	6 (16%); 4 (11%)
Lee et al. [15]	12 (10.3%); 0	43.8 ± 21.3; 35.8 ± 18.9	3 ± 0.39 (2–4); 3 ± 0.58 (2–5)	No significant difference at 12, 24, 36 h, and 14 days	4.0 (on 5-point scale); 3.3 (on 5-point scale)	17 (14.6%); 20 (17.7%)
Pan et al. [19]	0, 0	84.8 ± 25.1 (30–170); 77.9 ± 31.7 (30–170)	2.7 ± 0.6 (range: 2–5); 2.9 ± 0.9 (range: 2–5)	N/A	Higher satisfaction with scar appearance (TSILA); Lower satisfaction with 3-port	0, 3 (7.1%) → wound infection
Sozutek et al. [20]	0 (1 extra trocar used); 0	32.6 ± 9.9; 29.5 ± 6.8	1.1 ± 0.3; 1.2 ± 0.8	Pain Score VAS (3 h): 4.4 ± 1.1; 5.1 ± 1.2	Cosmetic Outcome Score (2–8): 7.2 ± 0.8; 6.7 ± 0.8	1 (4%); 1 (4%)
Teoh et al. [21]	8 (8.16%); 3 (3.09%)	63.0 ± 27.2; 60.2 ± 31.7	3.53 ± 2.92; 3.20 ± 2.36	Similar	Patient Satisfaction Score (VAS): 86.2 ± 17.6; 80.0 ± 24.4	14 (14.29%); 9 (9.28%)
Kim et al. [22]	0, 0	45.0 ± 15.5, 53.0 ± 32.8	1.2 ± 0.8; 1.6 ± 0.8	Pain Score (VAS) – at 6 h : 2.4 ± 1.9: 2.8 ± 1.4	N/A	0, 0
Kang et al. [23]	0, 0	43.8 ± 14.1; 29.3 ± 7.6	2.5 ± 0.9; 2.1 ± 0.7	N/A	Satisfaction – Wound Appearance: 77.4% “Very satisfied”; 68.8% “Very satisfied”	4 (4.4%); 2 (2.2%)
Assali et al. [24]		38.4 ± 19.0; 46.1 ± 19.2	2.0 ± 2.4; 1.9 ± 2.4			Ileus, Urinary Retention, Deep Space Infection, Readmission within 30 days

## Quality assessments

The cohort studies assessed via the Newcastle–Ottawa Scale show moderate quality, with consistent strengths in participant selection and outcome assessment, but limited comparability between groups. Liu et al. [5] rated highest (7/9), while the others scored 6/9, reflecting some methodological gaps (Table 2).

In contrast, the randomized studies generally exhibit a low risk of bias across key domains, particularly in outcome measurement and reporting. A few studies had minor concerns related to randomization or intervention adherence, but overall, the evidence base appears methodologically robust, especially for randomized designs (Fig. 2).

## Results

### Conversion to open surgery

**Fig. 3:**
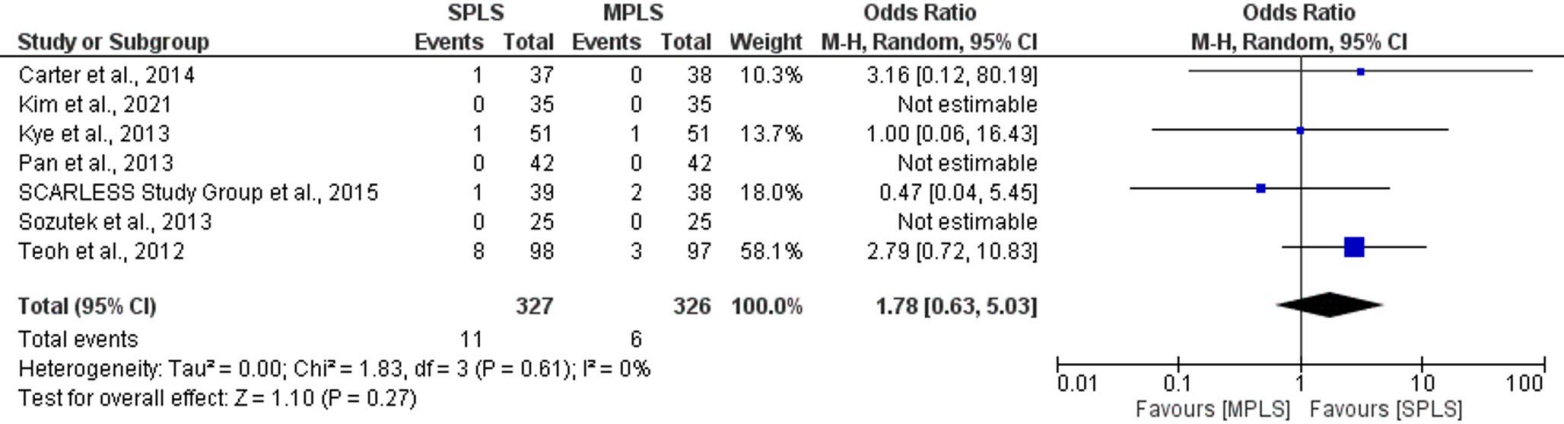
Forest plot comparing conversion rates to open surgery between SPLS and MPLS

The primary outcome was conversion rate from laparoscopic to open surgery. The pooled analysis demonstrated no statistically significant difference in conversion rates between the two groups (OR: 1.78; 95% CI: 0.63–5.03; *p = *0.27), Fig. 3. The total number of events was slightly higher in the SPLS group (11 of 327) compared to the MPLS group (6 of 326). Heterogeneity among studies was negligible (I^2^ = 0%), indicating consistent findings across trials (Fig. 3) [7, 17–22].

Several studies [19, 20, 22] could not be included in the OR estimation due to zero events in both arms [19, 20, 22]. Overall, the evidence suggests that SPLS does not significantly increase the risk of conversion to open surgery compared to MPLS.

### Operative time

**Fig. 4:**
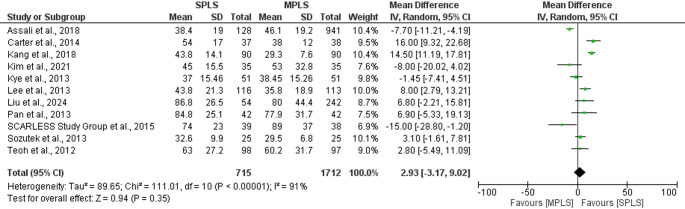
Forest plot comparing operative time between SPLS and MPLS

The overall mean difference in operative time was 2.93 min (95% CI: − 3.17 to 9.02; *p = *0.35). However, there was substantial heterogeneity among the studies (I^2^ = 91%, *p < *0.00001), likely reflecting variation in surgical experience, patient populations, and operative complexity, Fig. 4 [5, 7, 15, 17–24]. Overall, these findings suggest comparable operative times between SPLS and MPLS, though caution is warranted given the heterogeneity.

### Length of hospital stay

**Fig. 5:**
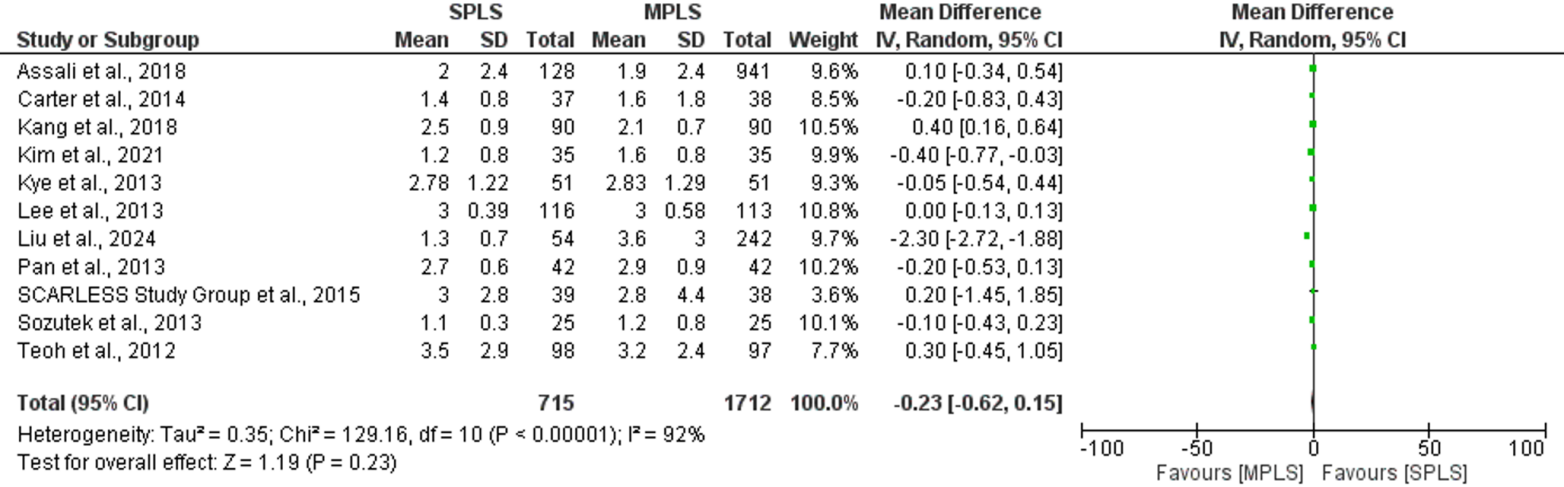
Forest plot comparing length of hospital stay between SPLS and MPLS

The pooled analysis showed no statistically significant difference in length of hospital stay between SPLS and MPLS (mean difference − 0.23 days; 95% CI: − 0.62 to 0.15; *p = *0.23). However, there was substantial heterogeneity among the studies (I^2^ = 92%; *p < *0.00001), likely due to variations in study populations, surgical techniques, and postoperative care protocols (Fig. 5) [5, 7, 15, 17–24]. Overall, these findings suggest comparable hospital stays between SPLS and MPLS, though the high heterogeneity indicates the need for cautious interpretation.

### Postoperative pain

**Fig. 6:**
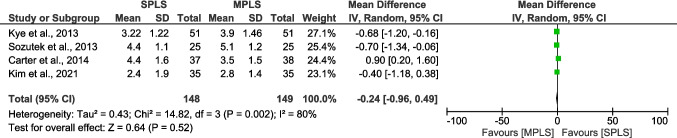
Forest plot comparing postoperative pain scores between SPLS and MPLS

The pooled mean difference reported that there was no statistically significant difference in pain scores between SPLS and MPLS (mean difference − 0.24; 95% CI: − 0.96 to 0.49; *p = *0.52). However, there was considerable heterogeneity between studies (I^2^ = 80%, *p = *0.002), which may be explained by differences in timing of pain assessment, analgesic regimens, and patient populations (Fig. 6) [7, 18, 20, 22].

### Cosmetic satisfaction

Cosmetic satisfaction following SPLS compared to MPLS has been reported in limited studies with considerable heterogeneity of measurement tools and assessment timing. Some utilized quantitative measures like Visual Analog Scales (VAS) 0 to 100, Likert-type scales (e.g., 1 to 5), and standardized body image questionnaires, while others provided qualitative descriptions of patient satisfaction.

For instance, SCARLESS Study Group et al., 2015 [17] reported significantly higher cosmetic satisfaction scores in the SPLS group on a validated cosmetic scale (mean 18.9 ± 4.1) compared to MPLS (mean 15.3 ± 5.8), reflecting better patient-graded scar appearance [17]. Similarly, Carter et al. [18] demonstrated higher satisfaction with scars in SPLS patients (mean 18.5 ± 2.2) versus MPLS (mean 16.9 ± 3.7) on a VAS scale, though this was not significant [18]. Pan et al. [19] also demonstrated improved cosmetic scores in SPLS on a 5-point Likert scale [19].

However, the timing of such measurements was highly variable, from early postoperative period to many months after surgery, which may affect patient perceptions according to scar maturation and accommodation. Further, the range of scales and lack of standardization of instruments render direct comparison and synthesis challenging.

### Surgical complication rate

**Fig. 7:**
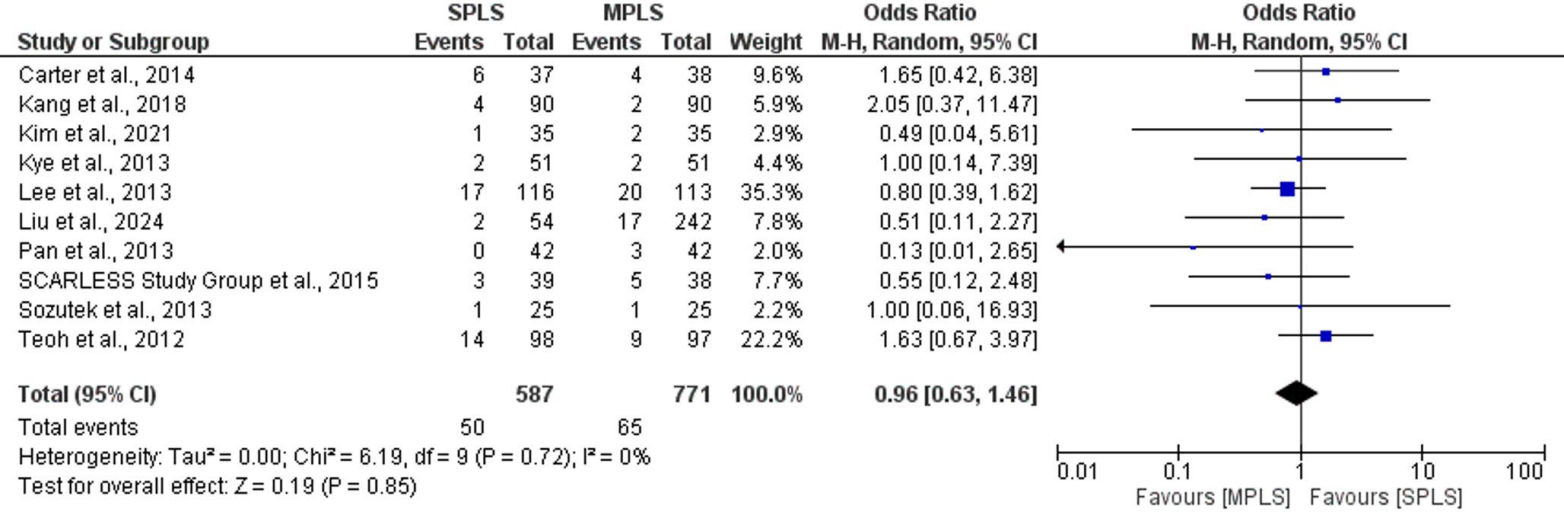
Forest plot comparing postoperative complication rates between SPLS and MPLS

Our meta-analysis showed no statistically significant difference in the complication rates between SPLS and MPLS, with a pooled odds ratio of 0.96 (95% CI: 0.63 to 1.46; *p = *0.85). There was no observed heterogeneity among the studies (I^2^ = 0%), indicating consistent results across trials (Fig. 7) [5, 7, 15, 17–23].

Assali et al. [24] reported postoperative complications in a large retrospective cohort comparing single-port laparoscopic appendectomy (SPLA) and multi-port laparoscopic appendectomy (MPLA) [24]. The study found no significant differences in rates of ileus, urinary retention, deep space infection, incisional hernia, or readmission between the two groups. However, this study was not included in the meta-analysis due to the absence of a clearly defined overall complication count, to avoid compromising the accuracy and consistency of the pooled analysis.

### Sensitivity analysis

**Fig. 8:**
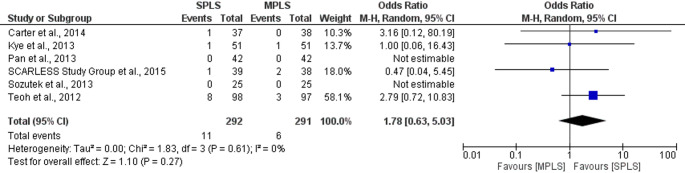
Forest plot of sensitivity analysis (RCTs only) comparing conversion rates to open surgery between SPLS and MPLS

To assess the robustness of our findings, sensitivity analyses were performed using only randomized controlled trials. For conversion rates to open surgery (*n* = 5), the pooled odds ratio was 1.78 (95% CI: 0.63–5.03; *p = *0.27), with no observed heterogeneity (I^2^ = 0%). Similarly, for postoperative complication rates (*n* = 8), no significant difference was found between SPLS and MPLS (OR = 1.04, 95% CI: 0.67–1.62; *p = *0.87; I^2^ = 0%). These findings closely mirrored the overall meta-analysis and suggest that the inclusion of retrospective studies did not introduce significant bias for these binary outcomes (Figs. 8 and 12).

**Fig. 9:**
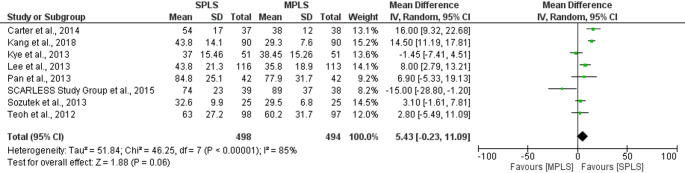
Sensitivity analysis (RCTs only) comparing operative time between SPLS and MPLS

**Fig. 10:**
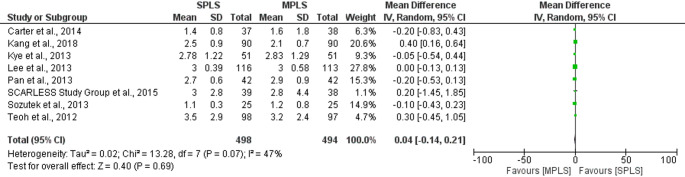
Sensitivity analysis (RCTs only) comparing length of hospital stay between SPLS and MPLS

**Fig. 11:**
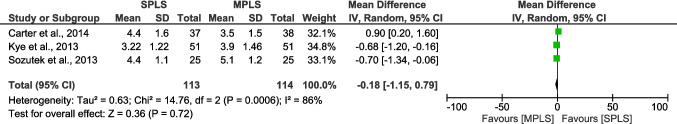
Sensitivity analysis (RCTs only) comparing postoperative pain scores between SPLS and MPLS

**Fig. 12:**
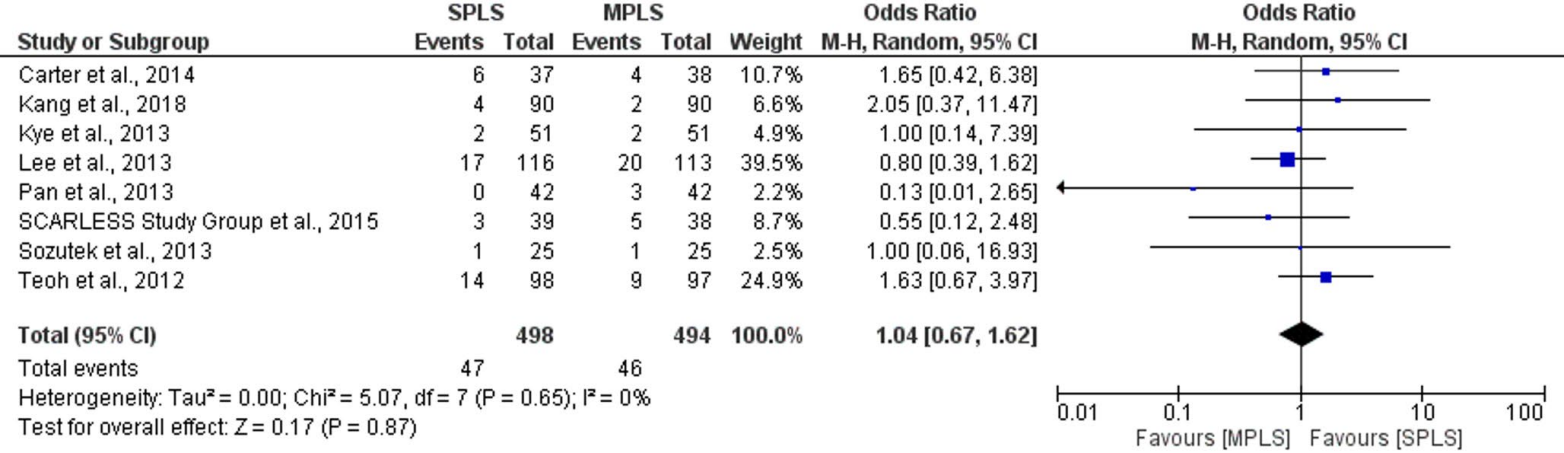
Sensitivity analysis (RCTs only) comparing postoperative complication rates between SPLS and MPLS

Among continuous outcomes, no statistically significant differences were found in operative time (mean difference = 5.43 min, 95% CI: − 0.23 to 11.09; *p = *0.06; I^2^ = 85%), length of hospital stay (mean difference = 0.04 days, 95% CI: − 0.14 to 0.21; *p = *0.69; I^2^ = 47%), postoperative pain scores (mean difference = − 0.18, 95% CI: − 1.15 to 0.79; *p = *0.72; I^2^ = 86%) or postoperative complication rates (mean difference= 1.04, 95% CI: 0.67 to 1.62; p= 0.87; I^2^ = 0%) when limiting analyses to RCTs (Figs. 9, 10, 11 and 12). High heterogeneity in operative time and pain scores likely reflects clinical and methodological variability across trials, such as differences in surgical expertise, analgesic protocols, and timing of assessments. Nevertheless, these results reinforce the consistency of findings across study designs and strengthen the reliability of the primary meta-analysis.

## Discussion

This systematic review and meta-analysis examined the comparative safety and efficacy of single-port laparoscopic surgery (SPLS) versus multi-port laparoscopic surgery (MPLS) for appendectomy. The findings indicate that, across a range of clinical outcomes including conversion rates, operative time, length of hospital stay, postoperative pain, and complication rates, SPLS performs similarly to MPLS. Although several studies reported marginal cosmetic benefits favouring SPLS, the lack of standardized measurement tools and timing limits the strength of this observation.

By restricting analyses to randomized controlled trials in a sensitivity analysis, we were able to explore the potential impact of study design on pooled outcomes. Notably, the exclusion of retrospective cohort studies did not materially alter the results, suggesting that the findings are robust across study types. Some outcomes, particularly operative time and postoperative pain, continued to exhibit substantial statistical heterogeneity. This likely reflects differences in surgical expertise, patient selection, and perioperative care protocols. Rather than diminishing the findings, these observations highlight the need for methodological standardization in future trials.

A strength of this review lies in its inclusion of recent trials published as late as 2024 and its comprehensive methodological approach, including adherence to PRISMA guidelines, prospective PROSPERO registration, and structured risk of bias assessments. In addition, the deliberately broad inclusion criteria allow this review to reflect the real-world diversity of SPLS practice across a variety of healthcare settings. This enhances external validity but may also contribute to the clinical heterogeneity observed across studies.

Despite these strengths, several limitations must be acknowledged. Many of the included trials had small sample sizes, limiting statistical power and increasing the risk of type two error. Inconsistent outcome definitions and measurement tools, particularly for pain and cosmetic satisfaction, further complicate direct comparison and pooling of data. Variability in the timing of outcome assessment also reduces the reliability of patient-reported endpoints. Moreover, few studies reported stratified data for key subgroups such as obese patients, older adults, or those with complicated appendicitis. As a result, subgroup analyses could not be conducted, representing a missed opportunity to explore differential treatment effects. Furthermore, possible selective outcome reporting and publication bias cannot be entirely ruled out. Future studies should prioritise stratified analyses of subgroups such as complicated versus uncomplicated appendicitis, sex, and BMI, which may reveal clinically meaningful differences in outcomes that are not apparent in pooled analyses.

In light of these limitations, the current evidence suggests that SPLS is a feasible and safe alternative to MPLS for appendectomy, with similar complication rates and recovery outcomes. However, due to persistent heterogeneity and modest trial quality in some areas, SPLS should not yet be considered equivalent to MPLS across all settings. Future large-scale, multicentre randomized trials using standardized outcomes and consistent reporting methods are essential to clarify the role of SPLS, particularly in high-risk or complex patient populations.

## Conclusion

While current evidence supports SPLS as a feasible and safe alternative to MPLS for appendectomy, the strength of this conclusion is limited by significant heterogeneity, small trial sizes, and variability in outcome measurement. Rather than suggesting equivalence, we advocate for further large-scale, multicentre randomized trials with standardized outcome definitions, especially to better assess patient-centred measures such as cosmetic satisfaction and recovery experience.

## Data Availability

No datasets were generated or analysed during the current study.

## Abbreviations

SPLS: Single-port laparoscopic surgery
MPLS: Multi-port laparoscopic surgery
SILA: Single-Incision Laparoscopic Appendectomy
CLA: Conventional Laparoscopic Appendectomy
LESS: Laparo-Endoscopic Single Site surgery
TPLA: Conventional Three-Port Laparoscopic Appendectomy
SPLA: Single-Port Laparoscopic Appendectomy
MPLA: Multi-Port Laparoscopic Appendectomy
MeSH: Medical Subject Headings
PRISMA: Preferred Reporting Items for Systematic Reviews and Meta-Analysis
AHRQ: Agency for Healthcare Research and Quality
PSM: Propensity score matching
VAS: Visual Analogue Scale
OR: Odds ratio
CI: Confidence interval
HR: Hazard ratio
SD: Standard deviation
